# Ngutulu Kagwero (agents of change): study design of a participatory comic pilot study on sexual violence prevention and post-rape clinical care with refugee youth in a humanitarian setting in Uganda

**DOI:** 10.1080/16549716.2021.1940763

**Published:** 2021-08-17

**Authors:** Carmen H. Logie, Moses Okumu, Simon Odong Lukone, Miranda Loutet, Alyssa McAlpine, Maya Latif, Isha Berry, Nelson Kisubi, Simon Mwima, Peter Kyambadde, Stella Neema, Eusebius Small, Senkosi Moses Balyejjusa, Joshua Musinguzi

**Affiliations:** a Factor-Inwentash Faculty of Social Work, University of Toronto, Toronto, ON, Canada; b Women’s College Research Institute, Women’s College Hospital, Toronto, ON, Canada; c Centre for Gender and Sexual Health Equity, Vancouver, BC, Canada; d United Nations University Institute for Water, Environment & Health (UNU-INWEH), Hamilton, ON, Canada; e School of Social Work, University of North Carolina Chapel Hill, Chapel Hill, NC, USA; f School of Social Work, University of Illinois at Urbana-Champaign, Urbana, IL, USA; g Uganda Refugee and Disaster Management Council, Yumbe, Uganda; h Dalla Lana School of Public Health, University of Toronto, Toronto, ON, Canada; i National AIDS Coordinating Program, Ugandan Ministry of Health, Kampala, Uganda; j Most at Risk Population Initiative (MARPI), Kampala, Uganda; k Department of Anthropology & Sociology, Makerere University, Kampala, Uganda; l School of Social Work, University of Texas Arlington, Arlington, TX, USA; m Department of Sociology and Social Administration, Kyambogo University, Kampala, Uganda

**Keywords:** Graphic medicine, sexual violence prevention, post-exposure prophylaxis, Uganda, refugee

## Abstract

With over 1.4 million refugees, Uganda is Sub-Saharan Africa’s largest refugee-hosting nation. Bidi Bidi, Uganda’s largest refugee settlement, hosts over 230,000 residents. There is a dearth of evidence-based sexual violence prevention and post-rape clinical care interventions in low- and middle-income humanitarian contexts tailored for refugee youth. Graphic medicine refers to juxtaposing images and narratives, often through using comics, to convey health promotion messaging. Comics can offer youth-friendly, low-cost, scalable approaches for sexual violence prevention and care. Yet there is limited empirical evaluation of comic interventions for sexual violence prevention and post-rape clinical care. This paper details the study design used to develop and pilot test a participatory comic intervention focused on sexual violence prevention through increasing bystander practices, reducing sexual violence stigma, and increasing post exposure prophylaxis (PEP) knowledge with youth aged 16–24 and healthcare providers in Bidi Bidi. Participants took part in a single-session peer-facilitated workshop that explored social, sexual, and psychological dimensions of sexual violence, bystander interventions, and post-rape clinical care. In the workshop, participants completed a participatory comic book based on narratives from qualitative data conducted with refugee youth sexual violence survivors. This pilot study employed a one-group pre-test/post-test design to assess feasibility outcomes and preliminary evidence of the intervention’s efficacy. Challenges included community lockdowns due to COVID-19 which resulted in study implementation delays, political instability, and attrition of participants during follow-up surveys. Lessons learned included the important role of youth facilitation in youth-centred interventions and the promise of participatory comics for youth and healthcare provider engagement for developing solutions and reducing stigma regarding SGBV. The Ngutulu Kagwero (Agents of change) project produced a contextually and age-tailored comic intervention that can be implemented in future fully powered randomized controlled trials to determine effectiveness in advancing sexual violence prevention and care with youth in humanitarian contexts.

## Background

Sexual and gender-based violence is a human rights violation and public health priority [1,2]. Sexual and gender-based violence is understood as any harm imposed on an individual on the basis of their gender and unequal power relationships [2]. Women and children living in humanitarian settings are disproportionately impacted by sexual and gender-based violence, with 21% of refugee women from 14 conflict-affected countries reporting experiences of sexual violence [2–4]. In humanitarian settings, factors that contribute to elevated exposure to community and intimate partner violence include the breakdown of community networks, reduced access to social and economic supports, increased poverty and food insecurity, changing family dynamics, and inequitable gender roles [4–7]. Violence is therefore produced across social-ecological levels. For instance, a 2017 systematic review that included 32 cross-sectional studies in humanitarian settings reported that exposure to household violence against women and children was associated with structural (low income), interpersonal (low social support), and intrapersonal (alcohol and substance use, poor mental health) level factors [8].

Systematic reviews of sexual and reproductive health interventions in low- and middle-income humanitarian contexts report limited sexual violence prevention and post-rape clinical care interventions focused on the lived experiences of youth [9,10]. Further, stigma and discrimination surrounding adolescents’ engagement in sexual practices and utilization of sexual and reproductive health (SRH) services, such as contraception and HIV testing, is associated with social isolation, violence, and mental health challenges [11,12]. Little is known of linkages between youth SRH stigma and uptake of post-rape care clinical services (e.g. post-exposure prophylaxis [PEP], emergency contraception) following sexual violence, particularly among refugee girls and young women [13,14].

Sexual violence stigma may also be associated with reduced uptake of post-rape clinical care [15–17]. For instance, in Kenya’s Dadaab Refugee Complex, adult women reported that sexual violence stigma from family and community members was a barrier to accessing sexual violence-related social and healthcare services [18]. Murray et al. [14] developed a scale to assess sexual violence stigma among adult women sexual violence survivors in the Democratic Republic of Congo, and found that sexual violence stigma was associated with higher depression, anxiety, and trauma symptoms. As sexual violence stigma was *not* associated with medical care seeking practices following sexual violence, study authors suggest that this may reflect the study sample’s prior engagement with psychosocial service providers and community-based women’s agencies [14]. Thus, the authors recommended further research in diverse contexts that also includes boys and men [14]. Little is known specifically of sexual violence stigma among refugee youth and its linkages to sexual violence prevention attitudes or post-rape clinical service uptake. Similarly, there are knowledge gaps regarding efficacious sexual violence stigma reduction strategies with refugee youth. Refugees may experience intersecting forms of stigma based on their refugee status, in addition to sexual violence stigma and gender inequities. An improved understanding of stigma experiences among refugee youth can inform tailored intervention strategies.

Numerous barriers contribute to knowledge gaps on efficacious sexual violence prevention and post-rape clinical care interventions for refugee youth [9,10,13]. These barriers include: 1) contextual diversity of socio-cultural, political, and religious values; 2) norms and beliefs within and between refugee populations and host countries; and 3) constrained financial and sexual health resources in humanitarian settings. Humanitarian funding largely needs to focus on crisis responses, and in turn there is little support for developing effective long-term sexual violence prevention strategies [13]. For example, UNHCR reported a 2020 funding gap of 64% for humanitarian programming in East and Horn of Africa and the Great Lakes, which reduced all facets of their responses, including protection, resilience, and solutions [19]. Recent studies conducted in the COVID-19 pandemic reveal concerning trends of increased sexual and gender-based violence and reduced access to SRH care in humanitarian contexts [20–23]. This includes concerns of increased child and forced marriages during the pandemic [21–24], which is already a prominent human rights concern affecting young refugee women who are sexual violence survivors [25–28]. Therefore, scalable and low-cost sexual violence prevention strategies are urgently needed for refugee youth during and following this pandemic.

Sexual violence prevention includes *primary prevention* to reduce the incidence of sexual violence and increase community wellbeing through changing underlying attitudes and practices that underlie violence perpetration. For instance, programs can challenge rape myths and sexual violence stigma and provide skills for bystander practices to interrupt or prevent violence [29]. *Secondary prevention* focuses on immediate responses following experiences of sexual violence, including addressing the direct consequences of violence in the short-term [29]. Finally, *tertiary prevention* involves long-term responses for persons who are sexual violence survivors, such as supportive services to manage long-lasting traumatic effects, in addition to programs for sexual violence perpetrators to prevent future violence [29]. The What Works violence prevention network describes various approaches to violence prevention interventions at the community, interpersonal, and intrapersonal levels [30]. The What Works strategies include community change agents, workshops and curriculum, working with community leaders, supporting state actors, and skills building [30]. They recommend group-based participatory approaches to learning, including reflection on gender norms, age-appropriate design, engaging and playful pedagogical approaches for youth, user-friendly manuals and materials [30], and support for sexual violence survivors. The What Works interventions have not yet been conducted with youth in refugee settlement contexts, and authors recommend acute sensitivity to local contextual factors, including drivers and facilitators of sexual violence [30]. The 2018 Interagency Field Manual Minimum Initial Service Package (MISP) for humanitarian contexts includes the objectives of sexual violence prevention and responding to survivors’ needs [31,32] and provides PEP administration instructions. The MISP also suggests engaging youth in generating solutions for sexual violence, engaging boys and men as positive agents of social change, gender-segregated programming for violence prevention, and community and healthcare engagement [32]. There is less focus in the MISP on youth-centered post-rape clinical care recommendations or strategies to enhance PEP knowledge and acceptability among refugee youth [32,33]. The MISP is focused on health providers, hence sexual violence recommendations center on health programming approaches [32]. Thus, our study aimed to build from this evidence base to develop contextually-, gender-, and age-tailored approaches to advance sexual violence prevention with refugee youth in a humanitarian setting.

Comics are an underexplored approach to advance sexual violence prevention, reduce sexual violence stigma, and increase PEP awareness and acceptability. The field of graphic medicine employs comics as a medium for patient care and education, using the juxtaposition of visual messages alongside text to convey emotions and underlying meaning [34]. Comics offer a youth-friendly, low-cost, scalable approach for providing education [35,36]. This approach is accessible, does not require high levels of literacy, and can engage persons in reflection on emotionally difficult and often stigmatized issues [37]. Comics have been used for health education [38] across varying health issues. For instance, the *Undetectables Intervention* included a comic book series as part of an intervention focused on increasing viral suppression among adults living with HIV in New York City [39]. Authors found an increase in viral suppression post-intervention that was attributed to increases in perceived worth and motivation regarding anti-retroviral therapy adherence [39]. Other approaches have used photo-novella strategies, whereby comic books utilize narrations with photos rather than illustrations. One such study with Latinx young adults aged 18–26 in Southern California found that the photo-novella (or fotonovella) was associated with increased human papillomavirus (HPV) perceived susceptibility and improved HPV vaccine acceptability, attitudes, and intention to use [40]. Fotonovellas have also been used as a health education tool that contributed to reductions in depression symptoms and mental health stigma among Latinx adults in Texas [41]. A psychoeducational comic book about depression, ‘*Somoud*,’ was pilot tested with conflict-affected adolescents in Lebanon [42]. Authors found that the comic held promise as an accessible, skills-focused, and engaging tool for learning about psychological reactions and coping [42]. Among refugee youth in Greece, a strengths-based narrative storytelling therapeutic intervention used a comic book as one of its methodologies and reported increased solidarity, goal setting, and critical thinking skills [43]. Another aim of comics in health education is to generate understanding and empathy about a particular health issue through sharing a memoir or lived experience [44]. For instance, the *Taking Turns: Stories from the HIV/AIDS Care Unit 371* [45] shared stories of a nurse’s experience providing care to people living with HIV alongside patient stories [46]. Taken together, the use of comics in graphic medicine holds promise in motivating behavioural change, increasing knowledge, and reducing stigma across a range of health issues, populations, and contexts. Yet, there is a limited evidence base of rigorously tested interventions using comics [42], particularly with refugee youth, that target sexual violence prevention, sexual violence stigma, PEP, and post-rape clinical care engagement.

This pilot study, *Ngutulu Kagwero* (loosely translated to ‘Agents of Change’ in Bari), aimed to address knowledge gaps regarding effective refugee youth-focused strategies for sexual violence prevention and post-rape clinical care [13] engagement using comics. We conducted a sequential transformative mixed-methods [47] study that involved qualitative data collection on sexual violence drivers and facilitators, followed by comic book development, and finally, the implementation of comic workshops with refugee youth and healthcare providers.

## Aims and objectives

This pilot study developed and tested the Ngutulu Kagwero intervention that aimed to reduce sexual violence stigma, increase bystander practices, and increase awareness and acceptability of post-rape clinical services among refugee youth in Bidi Bidi refugee settlement, Uganda [48,49]. The specific research objectives were to 1) assess the feasibility [50,51] of Ngutulu Kagwero through examining the process, resources, management, and scientific outcomes; and 2) to evaluate the effectiveness of Ngutulu Kagwero to change attitudes and behaviours towards sexual and gender-based violence and post-rape care among youth and healthcare providers in Bidi Bidi.

## Study design

**Figure 1.**
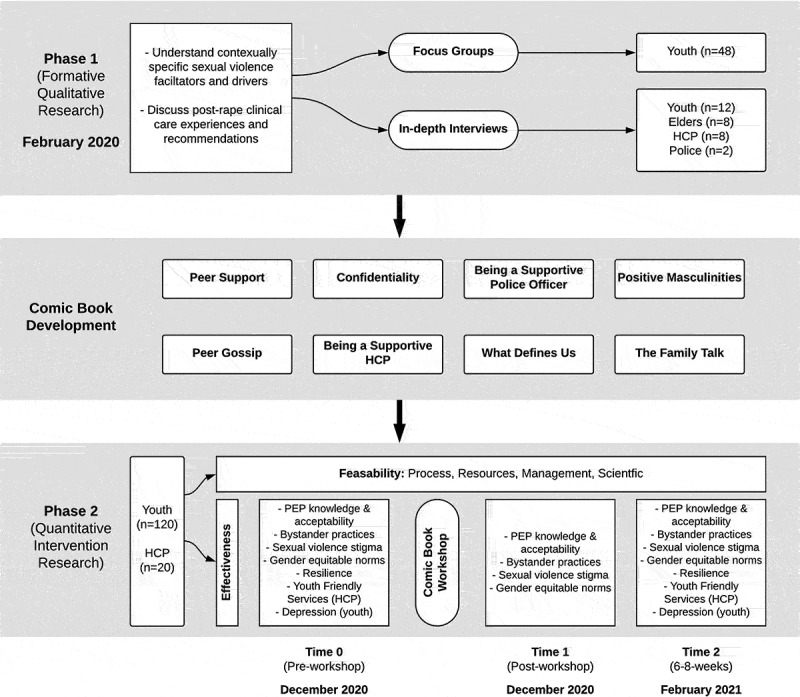
Flowchart of study phases and participant involvement

We conducted a sequential transformative mixed-methods [47] pilot study that involved a formative qualitative phase (February 2020) followed by a workshop (December 2020) with a pre-test (time 0) (December 2020), immediate post-test (time 1) (December 2020), and follow-up survey (6–8 weeks later) (time 2) (February 2021) (Figure 1). The formative phase involved conducting focus groups to generate knowledge of contextually-specific sexual violence drivers and facilitators, and in-depth individual interviews to explore lived experiences, with refugee youth aged 16–24, elders, and healthcare providers in Bidi Bidi. These findings were then used to create comic scenarios. The comic book included these completed scenarios, as well as blank scenarios, and were used in a workshop to share evidence-based skills and information. With the blank comics scenarios, youth had the opportunity to create solutions and scenarios for the comics. The workshop was evaluated using a single-group pretest post-test pilot study design. As a pilot study, the primary goal was to assess feasibility outcomes and preliminary evidence on the intervention’s effectiveness. Pilot studies ‘are an almost essential prerequisite’ in informing the design and success of future large, full-scale randomized controlled trials [50].

### Study setting

The study took place in Bidi Bidi Refugee Settlement within Yumbe district in northwestern Uganda. Established in September 2016, Bidi Bidi reached its capacity by December of the same year [52]. With over 232,000 refugees from South Sudan, Bidi Bidi is the second largest refugee settlement in the world [53]. Recent statistics from UNHCR report that women and children account for 86% of Bidi Bidi’s population, while young people between the ages of 15–24 comprise 22% of the overall population [53]. Challenges facing residents of Bidi Bidi include limited food and water supply, few academic institutions, overcrowded communities, scarcity of medical supplies, and little access to mental health services, which converge to elevate exposure to sexual violence and reduce access to post-rape clinical care [52]. Data from UNHCR indicates that in 2019 there were 4452 reported cases of sexual and gender-based violence in refugee settlements located in Uganda; however, there is likely underreporting due to sexual violence stigma [54]. Of these cases, 87% of survivors identify as women, and 14% of total cases involved children. The most commonly reported forms of violence were physical assault (30%), rape (26%), and psychological/emotional abuse (25%) [54]. Forced and early marriage accounted for 3% of overall reported sexual and gender-based violence cases in 2019 and is of particular concern in Bidi Bidi, where high school dropout rates have been reported amongst girls [54]. This study was implemented in Zone 3 of Bidi Bidi refugee settlement that has the largest population (*n* = 55,333) of Bidi Bidi’s five zones and is where the study partners at Uganda Refugee and Disaster Management Council work.

### Study population

We recruited a total of 220 participants in this mixed-methods study. In the qualitative Phase 1, we conducted in-depth interviews with: 12 refugee youth (youth in this study defined as ages 16–24) who were sexual violence survivors (female, *n* = 6; male, *n* = 6); eight refugee elders aged 55+ years old and/or persons identified as an elder by their community (female, *n* = 4; male, *n* = 4) aged 55+ ; eight healthcare providers (age 18+; female, *n* = 5; male, *n* = 3); and two police officers (age 18+; male, *n* = 2) in Bidi Bidi (Figure 1). Additionally, we conducted six focus groups with 48 refugee youth; three groups were conducted with young women (*n* = 24) and three groups with young men (*n* = 24). Interviews explored personal experiences and perspectives, and focus groups explored sexual violence drivers and facilitators, protective factors, and recommendations for prevention and for post-rape clinical care (Figure 1).

Phase 2 included refugee youth and healthcare providers. Participants were welcome to join Phase 1 and/or Phase 2. Inclusion criteria for refugee youth workshop participants included persons who: were aged between 16 and 24; identified as refugees or forcibly displaced persons; resided in the Bidi Bidi refugee settlement in Zone 3; were capable of providing informed consent; and spoke Juba Arabic, Bari, or English. Inclusion criteria for healthcare provider workshop participants included: persons aged 18 and older; providers of healthcare services in Bidi Bidi and/or to refugees in Yumbe; capable of providing informed consent; and spoke Juba Arabic, Bari, or English.

### Participant recruitment

We used purposive, non-random sampling methods [55] for participant recruitment. We engaged eight youth peer navigators (female, *n* = 4; male, *n* = 4), who identify as refugees and sexual violence survivors living in Bidi Bidi to facilitate recruitment alongside our collaborators working at health clinics and refugee agencies in Yumbe and Bidi Bidi. Peer navigators shared information about both study phases by word of mouth across their social networks and in spaces where youth congregate in the settlements. The peer navigators provided participants with referrals for sexual violence support in Bidi Bidi. Community collaborators also shared information about the study with youth, elders, healthcare providers, and community leaders in Zone 3. For healthcare providers, we similarly used purposive and convenience sampling [56] by collaborators who shared study information by word of mouth to recruit participants. Each participant was provided with cash honorariums: 1) to compensate for childcare and transportation for Phase 1; 2) an honorarium alongside food and refreshments during the workshops (including the preceding pre-test and immediate post-test) (Phase 2); and 3) at the follow-up survey for transportation and their time (Phase 2 follow-up survey).

### Intervention description

This study included Phase 1 (formative qualitative research) followed by Phase 2 (intervention, pre-test, post-test, follow-up survey) (Figure 1). In Phase 1, the study team trained youth peer navigators in research methods, including confidentiality. Qualitative research was conducted with refugee youth sexual violence survivors, elders, healthcare providers, and police officers to understand sexual violence drivers and facilitators (focus groups) and post-rape clinical care experiences and recommendations (in-depth interviews). The research team analyzed these qualitative data and worked together to develop scenarios for the comics [57].

### Participatory comic book intervention

The Ngutulu Kagwero comic book intervention was developed from thematic analysis [58,59] of qualitative data by the research team. Thematic analysis is a theoretically flexible analytic approach that involves six phases: 1) translating and transcribing the data, reading, and becoming familiar with the data; 2) developing codes across the entire data set with preliminary ideas; 3) exploring the ways that codes may reflect larger themes; 4) mapping the relationships between themes; 5) refining the themes and the overall story being generated by the data, including defining the themes; and 6) producing a report of the data by extracting selected data to reflect the themes, and tying the themes back to the existing literature [59]. We created eight scenarios for the comic book based on the emerging themes and findings from the analysis of qualitative data [57] that explored sexual violence experiences among youth and post-rape care preferences (Table 1). Each scenario is 1–2 pages in length in the comic book. The first draft of the scenarios was reviewed by peer navigators (*n* = 8), and study collaborators shared the scenarios with nine community members, including elders (*n* = 3), healthcare providers (*n* = 4), and police officers (*n* = 2) in Bidi Bidi. All feedback and suggestions were integrated into the final version of the comic book.

**Table 1.** Scenarios on sexual violence and post-rape clinical care included in the comic book

Scenario	Description
**Peer Support**	Explores strategies for peer support following a sexual violence disclosure, and how peers can encourage each other to seek post-rape healthcare services.
**Peer Gossip**	Presents the negative impact that gossip has on survivors of sexual violence and how to interrupt gossip when it happens.
**Confidentiality**	Illustrates the importance for HCPs to maintain confidentiality and how HCPs can champion a work environment that prioritizes confidentiality.
**Being a Support Health Care Provider**	Provides dialogue about PEP awareness and counseling strategies to be used by HCPs.
**Being a Supportive Police Officer**	Involves trauma-informed communication strategies to be used by police officers when working with sexual violence survivors.
**What Defines Us**	Challenges gendered and stigmatizing beliefs about sexual violence survivors by transforming these attitudes into a strengths-based and resilient narrative.
**Positive Masculinities**	Explores how men can be an active part of the solution in anti-violence initiatives in the community.
**The Family Talk**	Addresses forced and early marriage expectations and how sexual violence survivors can initiate dialogue with community members (e.g. family and elders) to challenge inequitable cultural and gender norms.

Each scenario in the comic book was accompanied by supplementary information or practice tips that further explored the illustrated content. Comics included panels with images/text of sexual violence scenarios. We also created a comic book with blank panels for participants to complete with their own thoughts and responses as part of the workshop.

For the comic book intervention itself, 120 youth participated in a one-day workshop (Table 2). We held a total of six workshops; three with 20 young women per workshop, and three with 20 young men per workshop. Healthcare providers (*n* = 20) also participated in a separate one-day workshop (Table 2). Workshop activities expanded on the content from the comic book scenarios. Each workshop began with ‘group norms’ activities to establish mutual respect and collaboration among group members [60].

**Table 2.** Workshop schedule and activity overview

Youth Workshop Activities	Healthcare Provider Activities
Participant introductions, consent forms, and pre-intervention surveys	Participant introductions, consent forms, and pre-intervention surveys
Group norms activity [60]	Group norms activity [60]
Activity 1: Understanding SGBV and its impacts [61] Activity 2: PEP knowledge and awareness [61,62]	Activity 1: Understanding SGBV and its impacts [61] Activity 2: PEP knowledge and awareness [61,62]
Activity 3: Reducing stigma towards SGBV survivors [61]	Discussion: Providing healthcare services to survivors of SGBV [62]
Activity 4: Introduction to bystander intervention and watching role-plays [63]	Activity 3: Reducing stigma towards SGBV survivors seeking health care [61]
Comic book reading and discussion	Activity 4: Introduction to bystander intervention and watching role-plays [63]
Comic book creation and sharing	Comic book reading and discussion
Closing circle	Comic book creation and sharing
Post intervention surveys	Closing circle
	Post intervention surveys

The youth workshop began with an interactive group activity to increase knowledge about sexual violence and the impact of sexual violence on women, men, children, family, and the community [61]. For healthcare providers, the group activity similarly focused on conceptualizing the impacts of sexual violence on youth, health, and the wider community. Additionally, we aimed to increase PEP knowledge and acceptability through providing information to both youth and healthcare providers about the utility of PEP, how it is administered, and the procedure for taking PEP [61,62]. Participants were also given a handout with this PEP information shared to take home with them. Healthcare providers engaged in additional discussions about post-rape healthcare preferences that emerged with youth in the qualitative phase, including youth recommended strategies to provide respectful, youth-tailored and non-discriminatory client care [62].

We also aimed to reduce sexual violence stigma through a group activity that involved a participant role-play illustrating sexual violence disclosure. The role-play instructed a participant to seek support from other group members, who were either peers (youth workshop) or healthcare provider colleagues (healthcare provider workshop) [61]. In the youth workshop, the first part of this activity involved group members responding ‘no’ to the participant seeking support to illustrate how sexual violence stigma impacts survivors and their ability to receive help and support [61]. In the healthcare provider workshop, the group member role-playing as a youth sexual violence survivor seeking health care is met with group members responding with unsupportive statements (e.g. ‘What did you do to have this happen to you?’), which illustrated how stigma and negative attitudes can occur within a healthcare setting. The second part of this role-play activity involved responding to the sexual violence disclosure with a supportive response (e.g. ‘I believe you’) to model ways to support sexual violence survivors, and in turn, to reduce sexual violence stigma. We provided handouts of supportive responses and each participant was given a supportive statement to practise.

Finally, we aimed to build knowledge and skills of prosocial practices across the continuum of violence by sharing information on bystander behaviours, informed by the Hollaback model of bystander intervention practices adapted with the study team for contextual relevance [63]. Youth and healthcare providers were given a SGBV scenario about encountering harassment while travelling home from collecting firewood. Participants were asked, in small groups, to formulate ideas on what to say to a harasser to intervene, how to ask for help from others, and what to do or say to support someone who experienced harassment. From the Hollaback model, these discussions encompass ‘direct,’ ‘delegate,’ and ‘delay’ strategies [63]. Additionally, youth and healthcare providers observed peer navigators facilitate three role-plays where scenarios about harassment were acted out and appropriate bystander interventions modelled, followed by a large group discussion. In both youth and healthcare provider workshops, role-plays examined how to intervene when witnessing harassment. These examples demonstrated bystander practices to address harassment while collecting firewood (youth example) and harassment at a bar (healthcare provider example). Specific to the youth workshop, another role-play explored how to intervene when witnessing a peer bullying a SGBV survivor. For healthcare providers, participants watched a second role-play about bystander intervention for addressing confidentiality breaches about SGBV survivors within the workplace.

In the second half of the workshop, the comic book was distributed among youth and healthcare providers (Table 2). The participants reviewed the content of the comic book scenarios with the workshop facilitators, and engaged in a group discussion about each scenario. After discussing the comic book as a group, each participant was invited to create their own responses for each scenario using the blank panels located in the comic book. There was an opportunity to share some participant responses within smaller groups and/or the larger workshop group. Participants completed a survey before, immediately after, and 6–8 weeks after the workshop to measure study outcomes (Figure 1).

### Sample size and power analysis

Phase 1 included 30 in-depth interviews with refugee youth sexual violence survivors (*n* = 12), refugee elders (*n* = 8), healthcare providers (*n* = 8), and police officers (*n* = 8) (Figure 1). Additionally, we conducted six focus groups that involved 48 refugee youth participants, including three focus groups with young women (*n* = 24) and three with young men (*n* = 24). While sample size in qualitative research is contested, a sample size of 25–30 individual interviews is often considered sufficient to realize saturation, to explore emergent concepts, and to explore diversity in experiences [64]. Focus groups are recommended to include approximately eight participants [65] per group, and researchers suggest conducting at least two focus groups per stratum (e.g. by gender). Therefore, our study included three focus groups per gender [66] in addition to in-depth interviews to generate sufficient data to reach saturation. Multimethod qualitative research that includes both focus groups and in-depth interviews can enrich understanding of study phenomena and offer additional validation of findings [67,68]. During Phase 2, 120 youth and 20 healthcare providers were recruited to participate in the comic book workshop with surveys conducted before, directly after, and six to eight weeks after the workshop. This pilot study’s primary focus was on feasibility, therefore not requiring a formal sample size calculation [50]. However, the sample size was sufficient to conduct paired sample t-tests with refugee youth data to explore preliminary effectiveness; the required sample size for this analysis is *n* = 109 as calculated with G*Power [69] (two-tailed, effect size: 0.35, *p* = 0.05, power 0.95, critical t: 1.98). The healthcare provider sample of 20 participants was included for feasibility and not powered to conduct pre- and post-test analyses.

### Outcomes

The primary outcomes to measure feasibility [51] of the Ngutulu Kagwero intervention were: 1) *process*, including recruitment rates; 2) *resources*, including retention rates, adherence, comprehension of surveys, length of time for survey completion, management of eligibility criteria; 3) *management*, including time and capacity to implement the intervention, equipment availability and functionality; and 4) *scientific outcomes*, including variability in the data, challenges entering surveys and following up with participants, and estimated effect of intervention participation on outcomes.

The outcomes to measure the potential effectiveness of the Ngutulu Kagwero intervention were integrated into the comic book scenarios and workshop content and included:

1. Changes in PEP knowledge and acceptability, assessed through testing participants on their knowledge of correct PEP use and acceptance towards PEP use and adherence [70–72]. To capture changes, the measure was assessed at all three study time points (pre-workshop [Time 0], post-workshop [Time 1], and 6–8 weeks [Time 2]).
2. Changes in self-reported bystander beliefs and behaviours, assessed through the Slaby Bystander Efficacy Scale [73] and Banyard et al., Readiness to Change Scale [74] (Time 0, Time 1, and Time 2).
3. Changes in attitudes and beliefs towards sexual violence, assessed through the Sexual Violence Stigma Scale [14] (Time 0, Time 1, and Time 2).
4. Changes in gender equitable norms, assessed using the physical violence subscale of the gender equitable men (GEM) scale [75] to measure attitudes towards gender equitable norms considering the prevailing norms in the community (Time 0, Time 1, and Time 2).
5. Changes in depression, assessed using the Patient Health Questionnaire 2-item (PHQ-2) [76]. This outcome was only measured among youth participants at Time 0 and Time 2.
6. Changes in the provision of youth-friendly services for youth who have experienced sexual and gender-based violence [77]. This outcome was only measured among healthcare provider participants at Time 0 and Time 2.
7. Changes in resilient coping to bounce back from challenging situations, assessed using the 4-item Brief Resilient Coping Scale (BRCS) [78] (Time 0 and Time 2).

### Data collection tools

Participants in the comic book workshops (*n* = 140) completed a tablet-based survey before participating in the intervention (Time 0), immediately following the intervention (later on the same day) (Time 1), and 6–8 weeks post intervention (Time 2).

### Data management and analysis plan

#### *Qualitative data (Phase 1*)

Focus group (FG) and in-depth interviews were digitally recorded and transcribed verbatim, and then translated from Bari and Juba Arabic into English. All transcripts were redacted (to remove any personally identifying information) and uploaded into Dedoose Version 8.0 data analysis software. We used thematic analysis [58,59] described above to analyze the focus group and in-depth interview data. Three researchers independently coded the data to increase reliability of results. Triangulation through member checking (local partners and peer navigators will provide feedback) and inviting multiple analysts to provide unique perspectives of the data increased the reliability and validity of findings. Themes that emerged from the qualitative data were used to inform the development of the comic book and intervention workshop in Phase 2.

#### *Quantitative data (Phase 2*)

Data were automatically uploaded onto a secure password protected software operated by SurveyCTO (Dobility, Cambridge, USA) [79]. SurveyCTO is a mobile data collection platform used on tablets or mobile phones to collect participant survey data at Time 0, Time 1, and Time 2. Data were collected in the field and encrypted as it was uploaded to the SurveyCTO project team server. All data were stored on password-protected computers. Descriptive analyses of socio-demographic variables, including means and standard deviations (SD), was conducted to provide an overview of participant characteristics. Secondly, items for each scale were summed to calculate overall and sub-scale scores, and we assessed scale reliability (e.g. calculating Cronbach’s alpha). Descriptive statistics were calculated to determine frequencies, means, and standard deviations for each summed score. We also assessed feasibility outcomes tracked on SurveyCTO, including length of survey completion and loss to follow-up to assess retention.

## Discussion

Our study addressed the lack of effective SGBV prevention and post-rape care interventions in humanitarian contexts tailored for youth [9,10] through developing and pilot-testing a comic book intervention, a promising strategy for increasing health promotion and literacy about stigmatized topics [37]. Comic books, and other forms of graphic medicine, have been used in interventions for a variety of health issues such as HIV, sexually transmitted infections, and dementia [80–84]. These tools can both educate the general population and be used to train healthcare providers to improve care and patient experiences [85]. Comics are particularly appropriate for youth as they provide health education in a fun, creative, and less intimidating format compared to traditional health information. Comics can also provide participants with the opportunity to view a situation from a different perspective and increase self-awareness [85,86]. In sum, comics are a promising yet understudied approach for improving health literacy of complex topics that can be difficult to discuss openly, such as sexual and gender-based violence [38,87].

This study was unique because it demonstrated how survivor-informed comic books can be used for both training youth on SGBV prevention strategies, and for training healthcare providers on how to respond to youth sexual violence survivors. Data cleaning and analyses are presently underway. The study process yielded insights and lessons learned from debriefing with the study team. First, a key component of success was engaging refugee youth along the research continuum: from sharing insights in the qualitative phase that were used to develop the original scenarios, to facilitating recruitment and implementation as peer navigators, and to engaging as participants that produced their own responses to violence in the comic scenarios. Peer navigators were positive role models, acquired research skills through the research process, and became recognized as knowledge holders in their community. Second, the community-based approach involved engaging with a local community-based agency along the research process; this approach facilitated recruitment, engagement, and retention of participants even with study delays and interruptions (detailed below). Collaborators were also fluent in local languages, had pre-existing knowledge and linkages with community members from offering support services and programs in Bidi Bidi, and had networks with other health and support services in Bidi Bidi that they were able to connect to study participants with as needed. For instance, the workshops were held in collaboration with an agency providing mental health services, and participants could be referred to this agency if they needed additional mental health support. Third, collaborators and peer navigators noted that the comic book approach itself motivated youth to participate in the study, was effective at delivering information and raising awareness of SGBV, and engaged both youth and healthcare providers in generating solutions to address violence in their community. Acceptability of this approach was also evidenced by youth participants who asked the study team for additional comic books to share with their friends. Anecdotally, healthcare providers reported being motivated to create youth-friendly spaces in their clinics after attending the workshop.

Challenges encountered during the project were largely related to delays and interruptions in the study due to COVID-19. For example, community lockdowns that started in March 2020 resulted in project implementation interruptions and delays. The team’s inability to travel to Bidi Bidi required all training for the intervention and data collection to be conducted virtually, which in turn was challenging  at times due to unstable internet connectivity. There was a second study interruption in January 2021 due to concerns regarding political instability and election violence, resulting in delaying the planned 4-week follow-up surveys to 6–8 weeks. In turn, these delays necessitated further virtual research refresher training for research assistants and peer navigators. The combination of COVID-19 travel restrictions and business closures due to this political instability rendered it difficult to acquire technology (e.g. tablets) for follow-up surveys, causing further delays. While planning and implementing follow-up surveys, collaborators noted some attrition due to participants relocating from Bidi Bidi and challenges reaching youth due to extended school closures from COVID-19. Despite these challenges, the study provided an opportunity to develop and test a comic book intervention for addressing sexual and gender-based violence, demonstrated feasibility and acceptability, and provided lessons learned about the benefits of youth and community engagement and comic book approaches.

To conclude, this study approach has the potential to inform research, practice, and policy. Though the Domestic Violence Act was enacted into law in Uganda in 2010, there are currently no guidelines specifically targeting sexual and gender-based violence among children, youth, or forcibly displaced persons [88]. Also, there are no known national guidelines on how healthcare providers respond to rape. Study findings therefore have the potential to not only inform a larger, fully powered randomized controlled trial to test the effectiveness of a comic book intervention, but can also inform policies on how strategies such as comic books can be integrated in school health curricula for sexual and gender-based violence prevention. Our findings can also inform research, practice, and policy on PEP information provision with youth in humanitarian contexts, and on PEP delivery by healthcare providers for rape survivors [89], to better meet the needs of refugee adolescents and youth. A comic book intervention approach holds promise for meaningfully engaging youth and healthcare providers in humanitarian contexts in dialogue on sexual and gender-based violence prevention, care, and support.

## Paper context

There is limited knowledge about effective sexual violence prevention and post-rape clinical care interventions tailored for adolescents and youth in humanitarian settings. This study involves developing and pilot testing a comic book intervention to reduce sexual violence stigma, increase post-exposure prophylaxis awareness, and improve bystander responses among refugee adolescents and youth in Bidi Bidi settlement, Uganda. The project contributes to knowledge of comic interventions and contextually-tailored strategies to address sexual violence with refugee youth.
